# Magnetic resonance neurography and diffusion tensor imaging of the sciatic nerve in hereditary transthyretin amyloidosis polyneuropathy

**DOI:** 10.1007/s00415-023-11813-z

**Published:** 2023-06-17

**Authors:** Roberto Gasparotti, Alessandro Salvalaggio, Daniele Corbo, Giorgio Agazzi, Mario Cacciavillani, Alessandro Lozza, Silvia Fenu, Grazia De Vigili, Matteo Tagliapietra, Gian Maria Fabrizi, Davide Pareyson, Laura Obici, Chiara Briani

**Affiliations:** 1 Neuroradiology Unit, Department of Medical and Surgical Specialties, Radiological Sciences, and Public Health, University of Brescia and ASST Spedali Civili Hospital, P.Le Spedali Civili 1, 25123 Brescia, Italy; 2 Department of Neurosciences, University of Padova, Via Giustiniani 5, 35128 Padua, Italy; 3 Padova Neuroscience Center (PNC), University of Padova, Padua, Italy; 4 Neuroradiology Unit, ASST Santi Paolo e Carlo Hospital, Milan, Italy; 5 Data Medica Group, CEMES, Padua, Italy; 6 Amyloidosis Research and Treatment Center, Fondazione IRCCS Policlinico San Matteo, Pavia, Italy; 7 Rare Neurological Diseases Unit, Department of Clinical Neurosciences, Fondazione IRCCS Istituto Neurologico Carlo Besta, Milan, Italy; 8 Parkinson and Movement Disorders Unit, Fondazione IRCCS Istituto Neurologico Carlo Besta, Milan, Italy; 9 Department of Neuroscience, Biomedicine and Movement Sciences, University of Verona, Verona, Italy

**Keywords:** Magnetic resonance neurography, Diffusion tensor imaging, Transthyretin amyloidosis, ATTRv amyloidosis, Amyloidotic polyneuropathy

## Abstract

The therapeutic advance in hereditary transthyretin amyloidosis (ATTRv amyloidosis) requires quantitative biomarkers of nerve involvement in order to foster early diagnosis and monitor therapy response. We aimed at quantitatively assessing Magnetic Resonance Neurography (MRN) and Diffusion Tensor Imaging (DTI) properties of the sciatic nerve in subjects with ATTRv-amyloidosis-polyneuropathy (ATTRv-PN) and pre-symptomatic carriers (ATTRv-C). Twenty subjects with pathogenic variants of the TTR gene (mean age 62.20 ± 12.04 years), 13 ATTRv-PN, and 7 ATTRv-C were evaluated and compared with 20 healthy subjects (mean age 60.1 ± 8.27 years). MRN and DTI sequences were performed at the right thigh from the gluteal region to the popliteal fossa. Cross-sectional-area (CSA), normalized signal intensity (NSI), and DTI metrics, including fractional anisotropy (FA), mean (MD), axial (AD), and radial diffusivity (RD) of the right sciatic nerve were measured. Increased CSA, NSI, RD, and reduced FA of sciatic nerve differentiated ATTRv-PN from ATTRv-C and healthy subjects at all levels (*p* < 0.01). NSI differentiated ATTRv-C from controls at all levels (*p* < 0.05), RD at proximal and mid-thigh (1.04 ± 0.1 vs 0.86 ± 0.11 *p* < 0.01), FA at mid-thigh (0.51 ± 0.02 vs 0.58 ± 0.04 *p* < 0.01). According to receiver operating characteristic (ROC) curve analysis, cutoff values differentiating ATTRv-C from controls (and therefore identifying subclinical sciatic involvement) were defined for FA, RD, and NSI. Significant correlations between MRI measures, clinical involvement and neurophysiology were found. In conclusion, the combination of quantitative MRN and DTI of the sciatic nerve can reliably differentiate ATTRv-PN, ATTRv-C, and healthy controls. More important, MRN and DTI were able to non-invasively identify early subclinical microstructural changes in pre-symptomatic carriers, thus representing a potential tool for early diagnosis and disease monitoring.

## Introduction

Hereditary transthyretin amyloidosis (ATTRv amyloidosis; v for variant) is an adult-onset genetic disease caused by the accumulation of misfolded transthyretin protein in different organs [1, 2].

More than 150 mutations have been identified in association with a broad range of clinical manifestations including more commonly an adult-onset progressive axonal peripheral neuropathy and an infiltrative cardiomyopathy [3–5]. As new therapies have recently become available for the treatment of ATTRv amyloidosis, earlier diagnoses have become crucial for patients’ care, considering the frequent diagnostic delay of several years from the first symptoms [6–8].

The demand for disease biomarkers is emerging in order to identify early pathological signs in pre-symptomatic carriers (ATTRv-C) and to monitor the course of the disease [1] as well as the response to treatment [6–8] in symptomatic patients (ATTRv-PN). Recent studies have demonstrated the impact of advanced magnetic resonance (MR) techniques for the investigation of peripheral nerves in ATTRv [9–12]. Subtle nerve abnormalities detected at proximal levels with MR Neurography (MRN), such as increased CSA and signal intensity of the lumbosacral plexus roots and the sciatic nerves at the thigh may precede the clinical or neurophysiological demonstration of polyneuropathy and a quantitative approach seemed able to differentiate ATTRv-PN and ATTRv-C from healthy controls. Magnetization Transfer Ratio (MTR) has also proved to be useful in quantifying macromolecular changes in ATTRv-PN and differentiating between ATTRv-PN and ATTRv-C with high sensitivity and correlation with Neuropathy Impairment Score at Lower Limbs (NIS-LL) and neurophysiological tests [9]. These methods have been used by a single institution to demonstrate early changes in pre-symptomatic disease stages in different polyneuropathies; however, they require complex post-processing and need further tuning to become more time and cost-efficient[13].

Alternative MR techniques have been recently used for the investigation of peripheral nerves, such as Diffusion Tensor Imaging (DTI), which provides measures of some microstructural properties of the nerve, through quantitative parameters such as Fractional Anisotropy, FA), Mean Diffusivity (MD), Axial Diffusivity (AD), and Radial Diffusivity (RD) [14–16].

FA and MD are general measures of the microstructural organization, which are sensitive to pathological processes, such as demyelination, axonal loss, and inflammation. RD and AD reflect the preferential direction of diffusion, perpendicular and parallel to fiber orientation, respectively, and are potentially more specific markers of demyelination and axonal damage, respectively [17]

DTI has not been applied yet to the investigation of ATTRv and offers potential advantages compared to other MR techniques, as reference values for the different DTI parameters already exist for the main peripheral nerve trunks in normal subjects [18]. Another potential advantage is represented by DTI fiber tracking that is increasingly used for visual display of peripheral nerves course [19–25].

In the present study, we aimed at evaluating the sciatic nerve in a cohort of subjects with ATTRv at different stages of the disease through an extensive morphological and ultrastructural evaluation based on quantitative magnetic resonance imaging, in order to identify possible biomarkers useful for early diagnosis and monitoring of the disease.

Our hypothesis was that the potential effects of ATTRv amyloidosis will be more evident compared to previous studies through a novel approach based on quantitative evaluation of peripheral nerve tissue properties revealed by DTI.

## Patients and method

### Patient enrollment

This is a cross-sectional multicenter study in which genetically confirmed ATTRv-PN patients and pre-symptomatic carriers were recruited in four Italian neurological centers.

Inclusion criteria: ATTRv-PN patients aged > 18 years, genetically confirmed (regardless of the type of mutation) and pre-symptomatic carriers aged > 18 years with genetically confirmed *TTR* mutation (regardless of the type of mutation).

Exclusion criteria: patients with diabetes or other conditions that may involve the peripheral nervous system, such as previous chemotherapy, patients with *pace-makers* or metal devices that were not compatible with MRI or with claustrophobia, non-cooperative patients.

Most of the included patients belong to a larger cohort of patients affected by ATTRv who have been extensively investigated with nerve ultrasound and previously reported in [26–28] and represent the sub-group of patients who accepted to undergo also MRN evaluation.

Twenty subjects with a *TTR* gene mutation (13 men, 7 women, mean age 62.20 ± 12.04 years, range 46–88) were enrolled, including 13 patients with axonal polyneuropathy (ATTRv-PN) and 7 pre-symptomatic carriers (ATTRv-C). The recruitment started in May 2016 and ended in January 2021.

The study was conducted according to the guidelines of the Declaration of Helsinki and approved by the Institutional Review Board (NP 2463/2016), written informed consent was obtained from all participants.

A control group of 20 gender and age-comparable subjects (mean age 60.1 ± 8.27, range 47–76) was selected for the analysis of MR data.

### Clinical evaluation of neuropathy

Clinical evaluation included the medical history (age and symptoms at onset, age at diagnosis, duration of disease, symptoms related to polyneuropathy and/or the involvement of other organs), and ongoing therapies. The severity of PN in lower limbs was scored with the NIS-LL [29]. The overall score of NIS-LL can range from 0 (no alteration) to 88 (total impairment) and consists of several sub-scores obtained from the evaluation of the following items: reflexes (0–8 points), muscle strength (0–64 points) and sensitivity (0–16). The characteristics of the patients are summarized in Table 1.

**Table 1:** Demographic data, *TTR* gene mutation, and neurophysiological data

Patient	Age	Sex	ATTRv	Type	Age at onset	NIS-LL	PND	Treatment	Fibular MCV (mV)	Fibular DML (ms)	Fibular CMAP (mV)	Sural SCV (m/s)	Sural SNAP (μV)
1	53	F	Phe84Leu	ATTRv-C CCCC	–	–	–	–	50.0	4.0	7.4	54.0	18.0
2	55	F	Phe84Leu	ATTRv-C	–	–	–	–	51.0	2.8	7.0	50.0	33.0
3	49	M	Phe84Leu	ATTRv-C	–	–	–	–	52.0	3.5	11.0	46.7	13.5
4	54	M	Val50Met	ATTRv-C	–	–	–	–	47.0	3.9	7.0	61.0	3.0
5	60	F	Glu62Lys	ATTRv-C	–	–	–	–	48.0	3.3	10.0	67.0	16.0
6	88	M	Val50Met	ATTRv-C	–	–	–	–	38.8	4.2	10.8	38.3	4.4
7	51	F	Glu54Gln	ATTRv-C	–	–	–	–	48.3	3.15	4.7	57.4	33.5
8	55	F	Glu89Lys	ATTRv-PN	53	42	IIIa	Patisiran	36.2	5.5	0.7	0	0
9	57	F	Glu109Gln	ATTRv-PN	56	5	I	Tafamidis	50.0	3.6	3.0	51.0	13.0
10	50	M	Glu109Gln	ATTRv-PN	50	5	I	Patisiran	42.8	3.8	1.4	0	0.0
11	56	M	Glu109Gln	ATTRv-PN	52	32	II	Patisiran		5.3		45.0	2.0
12	84	M	Val50Met	ATTRv-PN	81	31	II	Patisiran	42.2	4.8	1.4	43.8	1.6
13	76	F	Phe84Leu	ATTRv-PN	73	17	II	Tafamidis	43.0	4.0	4.0	0.0	0.0
14	71	M	Ile88Leu	ATTRv-PN	67	6	I	Tafamidis	38.0	3.9	3.0	33.0	1.0
15	71	M	Phe84Leu	ATTRv-PN	58	39	IIIb	Patisiran	33.0	5.5	2.3	29.3	2.9
16	63	M	Val50Met	ATTRv-PN	63	20	1	Tafamidis	44.0	3.7	0.5	38.0	0.5
17	60	M	Val50Met	ATTRv-PN	53	50	IIIa	Patisiran	41.0	4.3	0.1		
18	46	M	Thr49Ala	ATTRv-PN	44	8	I	Tafamidis	40.0	5.2	0.3	64.0	5.0
19	75	M	Tyr78Phe	ATTRv-PN	72	36	IIIa	Inotersen	0.0	0.0		0	0
20	70	M	Val50Met	ATTRv-PN	65	56	IIIa	Patisiran	18.0	5.6	0	0	0

### Neurophysiological assessment

The neurophysiological evaluation was carried out in accordance with the guidelines of the American Academy of Neurology, American Association of Electrodiagnostic Medicine (Jablecki CK, 2002). Nerve conduction studies (NCV) included compound motor action potential (cMAP), motor conduction velocities (MCV) and distal motor latencies (DML) of fibular nerve, sensory nerve action potential (SNAP), amplitude and sensory conduction velocities (SCV) of sural nerves (Table 1). The skin temperature was maintained at ≥ 32 °C throughout the study. Polyneuropathy was defined in accordance with the criteria by England et al. [30].

### High-resolution MR neurography-DTI

ATTRv-C and ATTRv-PN were investigated at the Neuroradiology Unit of the University Hospital of Brescia with a 3 Tesla MR Unit (Skyra, Siemens, Erlangen, Germany) with the following protocol: (a) axial high-resolution 2D T2-STIR sequences (MRN) of the right sciatic nerve from proximal to distal thigh (TR/TE 5200/54 ms, voxel size 0.4 × 0.3 × 4 mm), 60 sections, no intersection gap, scan time 4 min 42 s, (b) DTI of the right sciatic nerve with a spin-echo echo-planar-imaging sequence, strong fat-suppression pulse, bipolar diffusion gradients (TR/TE 87/7.7; *b* values, 0 and 800 s/mm^2^ encoded in monopolar 20 directions, voxel size 1.88 × 1.88 × 4 mm), 60 sections, no intersection gap, scan time 8 min 51 s. Sections were acquired at identical positions for both sequences. The MRN parameters are comparable to previous studies and the net imaging time including survey scans was 15:13 min. An 18-channel body-array-coil (Siemens Healthcare) was used for imaging of the sciatic nerve.

### Analysis of MR neurography and DTI data

The images were uploaded to a PACS workstation. The cross-sectional area (CSA) and the signal intensity of the right sciatic nerve were measured at three levels in the MRN sections: proximal thigh (slices 52–59), mid-thigh (slices 32–39), and distal thigh (slices 11–19). The CSA was assessed with a manual drawing of a Region of Interest (ROI) along the perineurium of the right sciatic nerve by a neuroradiologist with 15 years of experience in the field, and blinded to clinical data. The calculation of normalized signal intensity (NSI) of the sciatic nerve was computed as the ratio between the nerve intensity and the mean signal intensity of the biceps femoris long head muscle. The value of CSA and signal intensity (SI) was averaged resulting in a single value for each level*.*

The analysis of DTI parameters was performed on a PACS workstation with the Diffusion tool, included in the Philips Intellispace Portal system (Philips, Eindhoven), through the following steps: (1) automatic calculation of DTI maps: Fractional Anisotropy, (FA), Mean Diffusivity (MD), Axial Diffusivity (AD) and Radial Diffusivity (RD); (2) identification and manual contouring of the right sciatic nerve on DWI trace-weighted images (*b* = 800) corresponding to the MRN sections at proximal, mid-thigh, and distal thigh; (3) automatic ROI transfer to DTI maps. In slices where the peroneal and tibial divisions of the sciatic nerve were clearly separated, two distinct ROIs (one for each division) were drawn and the average value of the FA, MD, AD, and RD values was considered as the reference value for that slice*.* The value of DTI parameters was averaged resulting in a single value for each level*.*

DTI tractography was performed using manual contouring of the sciatic nerve in two ROIs on DWI-trace-weighted images as an anatomic reference at proximal and midthigh. Average values of FA and MD were calculated along the entire course of the sciatic nerve.

### Statistical analysis

The subjects were divided into 2 subgroups ATTRv-PN and ATTRv-C, according to the presence of amyloidosis-related polyneuropathy or absence in pre-symptomatic carriers, and compared to healthy controls.

Taking into consideration the rareness of the disease we did not apply a power analysis to our study population.

The normality of the variables was tested using the Kolmogorov–Smirnov method and variance equality with the Levene test.

Group comparisons were performed using a one-way ANOVA univariate test with post-hoc analysis and correction for multiple comparisons (Bonferroni).

The Mann-Whiney *U* test was applied for the comparison of differences in neurophysiological tests between ATTRv-PNP and ATTRv-C.

The linear correlation between two variables was tested using Pearson's r when both variables were normal-distributed. The predicted significance level was set at *p* < 0.05.

Receiver operating characteristic (ROC) curves analysis was applied to CSA, NSI, ad DTI parameters to identify possible thresholds able to discriminate patients and pre-symptomatic carriers from controls.

Statistical analysis was performed using SPSS (version 27, IBM Inc., Chicago, IL, USA) and Prism 9 (GraphPad Software, La Jolla, CA, USA) software.

## Results

### Subjects

The most prevalent point mutations were Val50Met (6 subjects), Phe84Leu (5 subjects).

Of the 20 enrolled patients, 13 (53%, 10 males, 3 females, mean age 63.4 ± 10.8, age range 46–84) had an axonal polyneuropathy, with an average NIS-LL 20.56 ± 17.65 (range 5–56). Seven subjects were pre-symptomatic carriers of whom 3 women and 4 men (mean age 58.7 ± 13.4, age range 49–88). Subjects were identified as ATTRv-PN or ATTRv-C according to a nerve conduction study. In particular, neurophysiological findings of the two sub-groups demonstrated an axonal polyneuropathy or resulted unremarkable, respectively. ATTRv-C did not report symptoms of small fiber neuropathy.

### Cross-sectional area (CSA) and normalized signal intensity (NSI) of the sciatic nerve

**Fig. 1:**
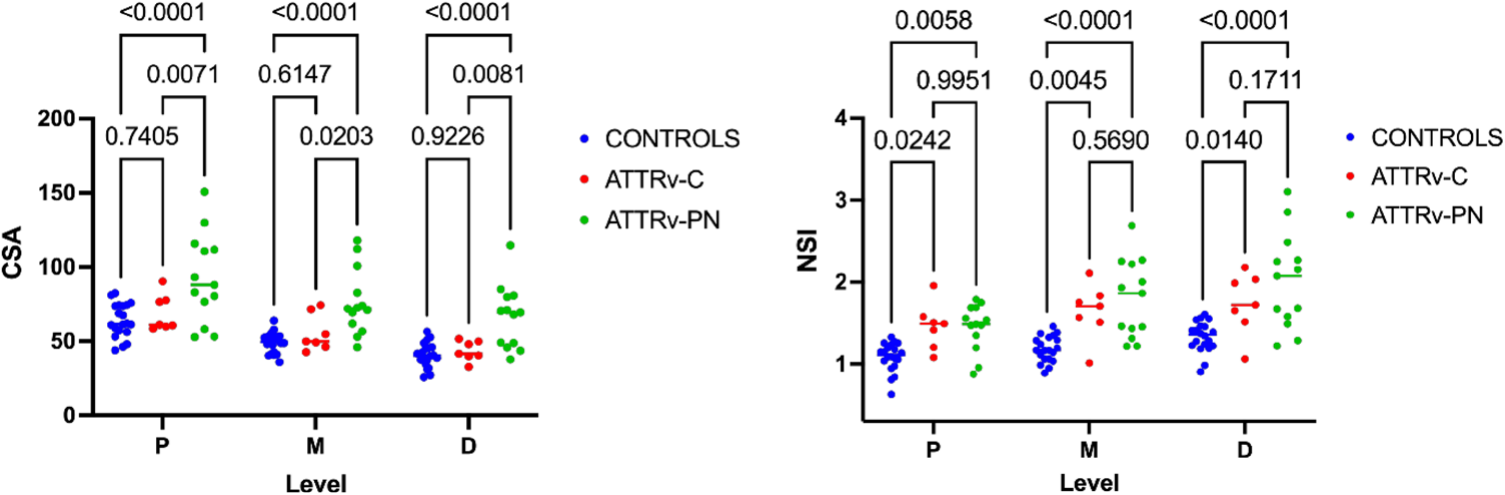
Quantitative MRN markers. Mean and SD values of sciatic nerve cross-sectional area (CSA) and normalized signal intensity (NSI) are plotted for controls, ATTRv-C, and symptomatic ATTRv-PN patients at proximal (P), mid (M), and distal (D) thigh. Sciatic nerve CSA was higher in symptomatic ATTRv-PN patients, with no significant differences between ATTRv-C and controls. NSI was significantly increased in ATTRv-C and ATTRv-PN compared to healthy controls, with no significant difference between ATTRv-C and ATTRv-PN. Significant differences are indicated by *p* values

The results are reported in Table 2, based on the anatomical level. CSA and NSI of the sciatic nerve were significantly different among the three groups along the thigh (one-way ANOVA 0.0001 < *p* < 0.01, *F* = 8.65–15.34). Mean CSA at proximal, mid-thigh, and distal thigh was slightly increased in ATTRv-C and significantly increased in ATTRv-PN compared to healthy controls (+ 44.5% proximal, + 55.7% mid-thigh, + 63.1% distal thigh). Mean NSI was significantly increased in ATTRv-C (+ 35.1% proximal, + 38.9% mid, + 30% distal) compared to controls and further increased in ATTRv-PN (+ 33.1% proximal, + 51.6% mid, + 33.5% distal). According to post-hoc analysis, ATTRv-PN had significantly increased CSA and NSI of the sciatic nerve along the whole thigh compared to healthy controls (*p* < 0.0001) and significantly increased CSA at proximal (*p* < 0.01), mid (*p* < 0.05) and distal thigh (*p* < 0.01) compared to ATTRv-C (Fig. 1). No significant differences of sciatic nerve CSA were found between ATTRv-C and healthy controls at the three levels. ATTRv-C had significantly increased NSI along the whole course of the sciatic nerve at the thigh (0.001 < *p* < 0.05) compared to controls. No significant differences of NSI were found between ATTRv-PN and ATTRv-C.

**Table 2:** Results of quantitative MRI assessment of sciatic nerves at the proximal, mid, and distal thigh

Sciatic nerve at thigh	Controls	ATTRv-C	ATTRv-PN	*p*
Proximal				
CSA	64.13 ± 11.38	69.37 ± 12.36	92.73 ± 30.06	< 0.001
NSI	1.08 ± 0.17	1.46 ± 0.28	1.44 ± 0.28	< 0.001
FA	0.58 ± 0.02	0.54 ± 0.03	0.46 ± 0.05	< 0.001
MD	1.37 ± 0.16	1.49 ± 0.13	1.66 ± 0.22	< 0.01
AD	2.26 ± 0.26	2.49 ± 0.23	2.42 ± 0.25	n.s
RD	0.86 ± 0.11	1.04 ± 0.1	1.22 ± 0.18	< 0.001
Mid				
CSA	48.93 ± 6.85	55.61 ± 12.42	76.22 ± 22.04	< 0.001
NSI	1.18 ± 0.15	1.64 ± 0.33	1.79 ± 0.47	< 0.001
FA	0.58 ± 0.04	0.51 ± 0.02	0.41 ± 0.05	< 0,001
MD	1.41 ± 0.17	1.58 ± 0.14	1.8 ± 0.22	< 0.001
AD	2.38 ± 0.28	2.49 ± 0.24	2.47 ± 0.14	n.s
RD	0.91 ± 0.16	1.12 ± 0.15	1.35 ± 0.15	< 0.001
Distal				
CSA	40.77 ± 8.17	43.48 ± 6.07	66.53 ± 21.4	< 0.001
NSI	1.33 ± 0.18	1.73 ± 0.37	2 ± 0.58	< 0.001
FA	0.57 ± 0.04	0.53 ± 0.04	0.42 ± 0.05	< 0.001
MD	1.41 ± 0.14	1.48 ± 0.23	1.73 ± 0.22	< 0.001
AD	2.36 ± 0.21	2.25 ± 0.24	2.39 ± 0.19	n.s
RD	0.92 ± 0.14	0.98 ± 0.17	1.28 ± 0.21	< 0.001
Mean NSI	1.19 ± 0.14	1.61 ± 0.28	1.75 ± 0.4	< 0.001
Tractography mean FA	0.56 ± 0.03	0.53 ± 0.04	0.42 ± 0.04	< 0.001
Tractography mean MD	1.36 ± 0.23	1.55 ± 0.17	1.71 ± 0.22	< 0.001

The combination of CSA and NSI of the right sciatic nerve was able to classify the 3 subgroups as follows: ATTRv-PN patients have increased CSA and NSI along the entire thigh, whereas ATTRv-C has nearly normal CSA and increased NSI compared to healthy controls.

### Diffusion tensor imaging (DTI) of sciatic nerve

DTI metrics of the sciatic nerve, according to the anatomical level, are reported in Table 2.

All sciatic nerve DTI parameters, but AD, were significantly different among the three groups at all levels (one-way ANOVA *p* < 0.0001, *F* = 9.6–60.9).

Reduced FA and increased MD and RD differentiated ATTR-PN from ATTRv-C and controls at all anatomical levels (*p* < 0.01).

As from the post-hoc analysis, FA was significantly reduced at mid-thigh in ATTRv-C compared to controls (0.51 ± 0.02 vs 0.58 ± 0.04 *p* = 0.001), whereas RD was significantly increased at proximal (1.04 ± 0.1 vs 0.86 ± 0.11 *p* < 0.001) and mid-thigh (1.12 ± 0.15 vs 0.91 ± 0.16 *p* < 0.01). In ATTRv-PN FA was significantly reduced (*p* < 0.0001) and RD significantly increased (0.001 < *p* < 0.05) compared to ATTRv-C at all levels. Mean FA and MD values derived from DTI tractography of the sciatic nerve were able to differentiate ATTRv-PN from controls (FA 0.42 ± 0.04 vs 0.56 ± 0.03 *p* < 0.0001, MD 1.71 ± 0.22 vs 1.36 ± 0.22 *p* < 0.0001, TL 65 ± 26 vs 109 ± 49 *p* < 0.0001), mean FA was able to differentiate ATTRv-PN from ATTRv-C (FA 0.42 ± 0.04 vs 0.53 ± 0.04 *p* < 0.01), whereas mean FA and mean MD were unable to differentiate ATTRv-C from controls.

Tractography of the right sciatic nerve was able to display the whole course of the sciatic nerve from proximal to distal thigh in all cases.

**Fig. 2:**
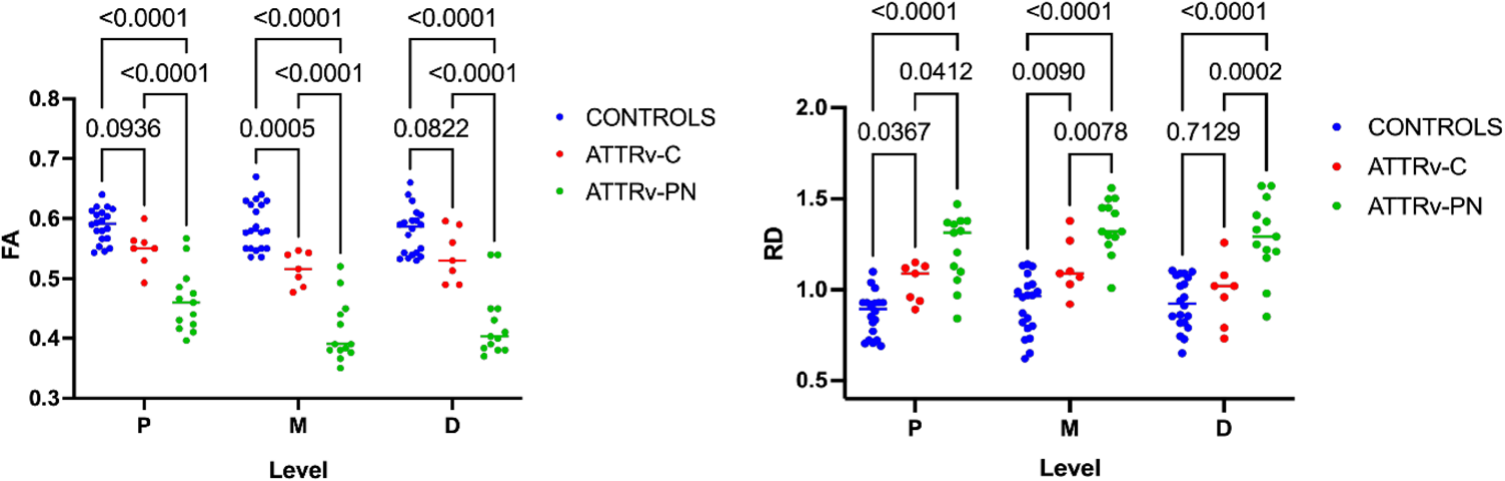
Quantitative DTI metrics. Mean and SD values of sciatic nerve Fractional Anisotropy (FA) and Radial diffusivity (RD) are plotted for healthy controls, ATTRv-C and ATTRv-PN patients at proximal (P), mid (M), and distal (D) thigh. Sciatic nerve FA reached the highest values in healthy controls, decreased in ATTRv-C, and further decreased in ATTRv-PN patients. Sciatic nerve RD was highest in ATTRv-PN patients and decreased in pre-symptomatic carriers, reaching the lowest values in healthy controls. FA at mid-thigh and RD at proximal and mid-thigh were able to differentiate pre-symptomatic carriers from healthy controls. Significant differences are indicated by *p* values

The boxplots of FA and RD values at proximal (P), medium (M), and (D) distal sciatic nerve in ATTRv-PN, ATTRv-C, and healthy controls are reported in Fig. 2.

**Fig. 3:**
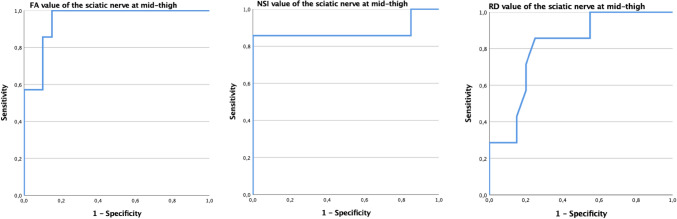
Receiver operating characteristic (ROC) curves of the sensitivity and specificity of DTI values (FA and RD) and NSI in the sciatic nerve at mid-thigh to distinguish ATTRv-C from controls

The ROC curve analysis showed that the cutoff value of sciatic nerve FA at mid-thigh able to differentiate ATTR-C from controls was 0.54, with 100% sensitivity, 85% specificity, and area under the ROC curve (AUC) of 0.95 (*p* < 0.001). The cutoff value of NSI was 1.48 with 85% sensitivity, 100% specificity, and AUC of 0.879 (*p* < 0.01); the cutoff value of RD was 1.02 with 85% sensitivity, 75% specificity, and AUC of 0.814. (*p* = 0.01) (Fig. 3).

**Fig. 4:**
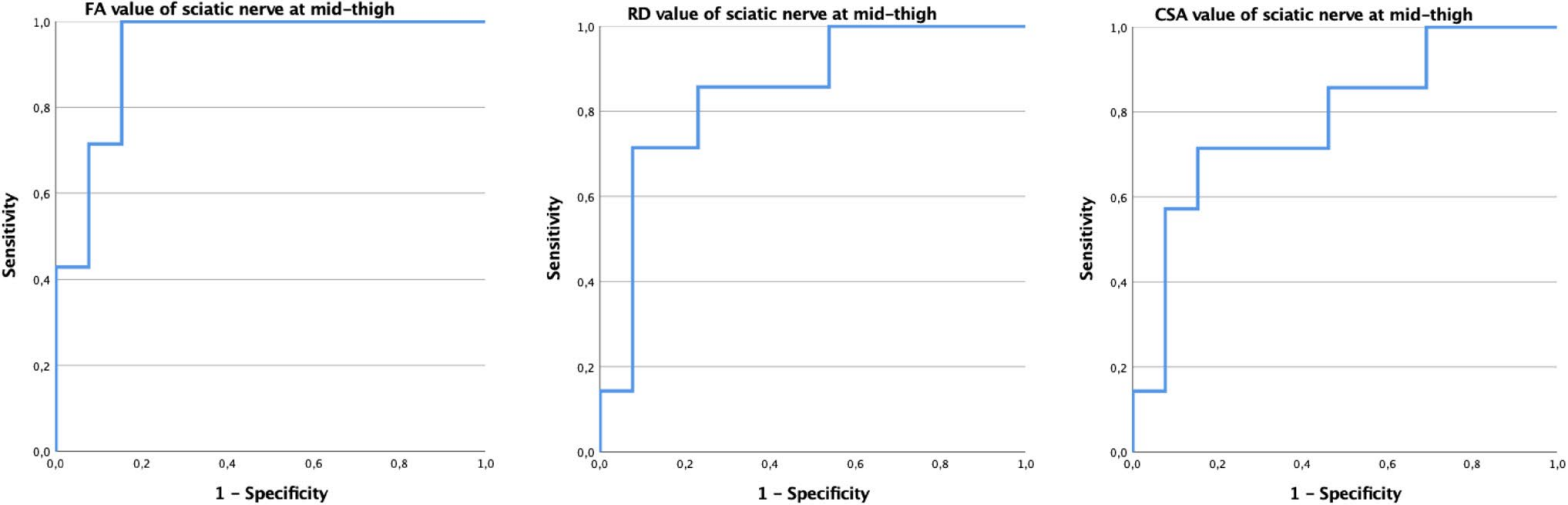
Receiver operating characteristic (ROC) curves of the sensitivity and specificity of DTI (FA and RD) and CSA of the sciatic nerve at mid-thigh, to distinguish ATTRv-PN from ATTRv-C

The cutoff value of sciatic nerve FA at mid-thigh able to differentiate ATTR-PN from ATTR-C was 0.48, with a sensitivity of 85%, 85% specificity and area under the ROC curve (AUC) of 0.934 (*p* < 0.01); the cutoff value of RD was 1.28 with 77% sensitivity and 85% specificity, and area under the ROC curve (AUC) of 0.84 (*p* = 0.01); the cutoff value of CSA was 55.76 mm^2^ with 85% sensitivity, 71% specificity, and area under the ROC curve (AUC) of 0.78 (*p* < 0.05) (Fig. 4).

In summary, according to our results, early signs of sciatic involvement in ATTRv-C can be defined by the combination of the following parameters measured at mid-thigh: FA < 0.54, RD > 1.02, NSI > 1.48.

The main correlations between imaging, clinical and neurophysiological findings are reported in Table 3.

**Table 3:** Summary of the relevant correlations between MR, clinical, and neurophysiological findings

	NIS-LL	MCV fibular	CMAP fibular	SCV sural	SNAP sural	CSA	NSI	FA	RD
CSA	M	P	D	*N.S*	D	*–*	D	D	D
	*r* = 0.744	*r* = − 0.906	*r* = − 0.651		*r* = − 0.507		*r* = 0.680	*r* =− 0.693	*r* = 0.699
	*p* < 0.0001	*p* < 0.001	*p* < 0.05		*p* < 0.05		*p* = < 0.0001	*p* = < 0.0001	*p* = < 0.0001
NSI	*N.S*	*N.S*	*N.S*	*N.S*	*N.S*	D	*–*	M	M
						*r* = 0.680		*r* = − 0.722	*r* = 0.711
						*p* = < 0.0001		*p* < 0.0001	*p* < 0.0001
FA	M	P	P	P	M	D	M	*–*	M
	*r* = − 0.775	*r* = 0.638	*r* = 0.824	*r* = 0.605	*r* = 0.708	*r* = − 0.693	*r* = − 0.722		*r* = − 0.941
	*p* < 0.0001	*p* < 0.01	*p* < 0.0001	*p* < 0.05	*p* = 0.001	*p* = < 0.0001	*p* < 0.0001		*p* < 0.0001
RD	M	P	M	*N.S*	D	D	P	M	*–*
	*r* = 0.722	*r* = − 0.582	*r* = − 0.841		*r* = − 0.630	*r* = 0.699	*r* = 0.711	*r* = − 0.941	
	*p* < 0.0001	*p* < 0.05	*p* < 0.0001		*p*< 0.01	*p* < 0.0001	*p* < 0.0001	*p* < 0.0001	
DTT mean FA	*r* = − 0.636	*r* = 0.566	*r* = 0.788	*N.S*	*r* = 0.673	M	M	*–*	M
						*r* = 0.628	*r* = − 0.732		*r* = − 0.859
	*p* < 0.01	*p* = 0.05	*p* = 0.001		*p* = 0.001	*p* = 0.0001	*p* = 0.0001		*p* < 0.0001

r   Pearson coefficient, P  proximal thigh, M   mid-thigh, D   distal thigh, DTT   Diffusion Tensor Tractography, N.S   non-significant

CSA of the sciatic nerve positively correlated with NIS-LL and RD, and negatively correlated with FA and cMAPs and MCVs of the fibular nerve.

NSI of the sciatic nerve positively correlated with RD and negatively with FA.

FA negatively correlated with NIS-LL, CSA, and NSI of the sciatic nerve and positively correlated with cMAPs and MCVs of the fibular nerve, and MCVs and SNAPs of the sural nerve.

RD instead positively correlated with NIS-LL and CSA and NSI of the sciatic nerve, and negatively with cMAPs and MCVs of the fibular nerve and MCVs and SNAPs of the sural nerve.

**Fig. 5:**
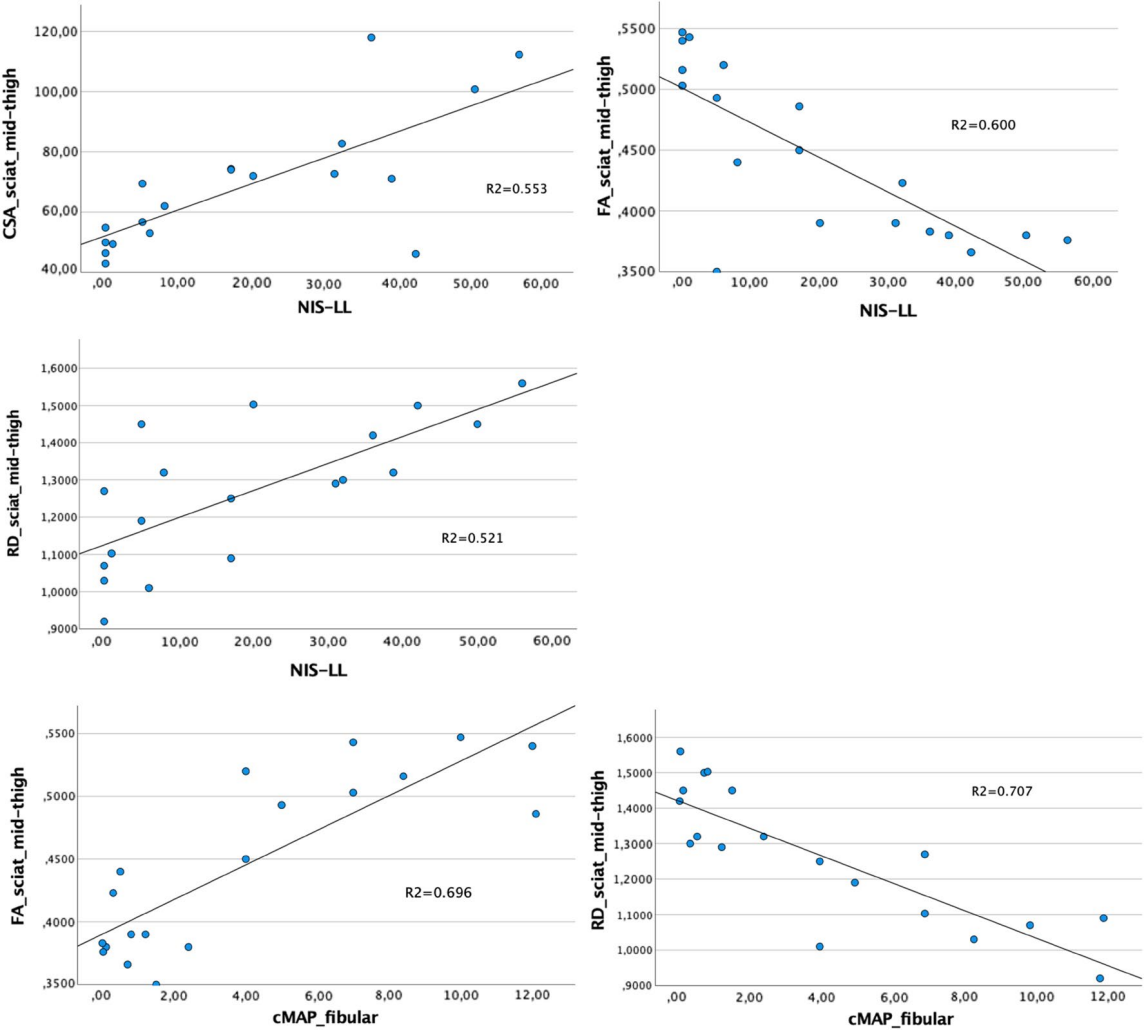
Scatter plots relating FA and RD of the sciatic nerve at mid-thigh and mean FA values derived from DTI tractography with nerve conduction studies of the fibular and sural nerve

Scatter plots relating DTI metrics (FA, RD) with nerve conduction studies are displayed in Fig. 5.

No significant correlations were found between NSI of the sciatic nerve and neurophysiological findings.

## Discussion

In the present study, we performed a comprehensive MR evaluation of the sciatic nerve in a cohort of ATTRv amyloidosis patients (both with polyneuropathy and pre-symptomatic carriers). We investigated nerve morphology (CSA), tissue integrity (NSI) and, for the first time in the ATTRv amyloidosis population, nerve ultrastructure with DTI (FA, MD, AD, RD). Moreover, correlations between imaging-derived features, neurophysiological measures (MCV, SCV, SNAP, and cMAP) and disease severity (NIS-LL) were assessed. The findings may be summarized as follows: imaging derived measurements were able to distinguish ATTRv-PN from pre-symptomatic carriers and controls and correlated with electrophysiology and clinical impairment.

A rapidly progressive, distal-symmetric, axonal, sensorimotor polyneuropathy is one of the main manifestations in ATTRv amyloidosis and leads to a significant decline in patients’ quality of life, affecting mobility, daily life activities, and the ability to work [31].

New treatments have become recently available [6, 7] thus significantly improving the prognosis of patients with ATTRv. Delay in starting treatment has been associated with a worse long-term prognosis, therefore early diagnosis or prompt identification of peripheral nerve involvement in pre-symptomatic carriers are crucial for starting timely therapy and preserving patients’ quality of life [3, 32]. In this scenario, the availability of a non-invasive imaging marker of peripheral nerve involvement may provide therapeutic and prognostic advantages.

To this aim previous studies have demonstrated the potential role of advanced magnetic resonance techniques in the early identification of ATTRv-polyneuropathy, proposing T2 relaxometry, proton density (*ρ*), and magnetization transfer ratio (MTR) as potential new imaging biomarkers of the disease. Nonetheless, the complexity of the measures prevented their implementation in clinical settings and the correlation with neurophysiological data has not been widely explored [9–12].

In the present study, we propose a simplified MRI multimodal approach based on MRN and DTI of the sciatic nerve, providing a combined quantitative assessment of morphological and ultrastructural features. Two easily measurable MRN morphological parameters such as CSA and normalized signal intensity (NSI) of the sciatic nerve at the thigh were combined with a quantitative assessment of DTI parameters (FA, MD, AD, RD), including tractography.

ATTRv-PN patients showed increased CSA and NSI of the sciatic nerve at the thigh compared to healthy controls, ATTRv-C had increased NSI and no significant changes in size compared with controls and ATTRv-PN had increased CSA compared with ATTRv-C and no significant changes in NSI.

In agreement with previous studies by Kollmer et al. [9–11] using NSI as an alternative to T2 relaxometry and proton density calculation, we were able to demonstrate that signal intensity changes in the sciatic nerve can differentiate ATTRv-C from healthy controls, although NSI was unable to differentiate ATTRv-C from ATTRv-PN. However, in our study, the CSA of the sciatic nerves was significantly larger in ATTRv-PN compared to controls; therefore, a combination of the two parameters can be used to differentiate the three subgroups.

CSA enlargement represents a confirmation of previous ultrasound and MRI findings (with an enlargement of nerves at proximal sites, such as brachial and lumbosacral plexi) [12, 26–28] and represents a peculiarity since increased CSA is usually associated with demyelinating polyneuropathies [33, 34]. According to pathological studies, proximal CSA enlargement may reflect the pattern of amyloid deposition which appears to be more pronounced proximally and a possible direct damage to Schwann cells by amyloid protein aggregates has been postulated [34].

The increased NSI of the sciatic nerve in ATTRv may be related to the impairment of the blood-nerve barrier which exposes the nerve to the toxic effects of TTR with consequent modifications of axoplasmic flow and increased water content. Similarly, a recent paper has demonstrated the ability of T2 relaxometry and proton spin density of the sciatic nerve to differentiate controls from patients with amyloid light chain (AL) polyneuropathy and mild and moderate disease severity [35].

As a novel finding compared with previous papers, our study showed DTI abnormalities in the ATTRv amyloidosis cohort with a progressive worsening from ATTRv-C to ATTRv-PN. In ATTRv-C the FA of the sciatic nerve was significantly decreased at mid-thigh and RD was significantly increased at proximal and mid-thigh compared to healthy controls. Further decrease of FA and increase of RD was observed in ATTRv-PN along the whole course of sciatic nerve at thigh, compared with ATTRv-C. Among MRN and DTI parameters, decreased FA at mid-thigh was the most reliable in differentiating ATTRv-C from controls and ATTRv-PN, with cutoff values which can be individually applied to improve the diagnostic accuracy. Notably, 100% of sensitivity was achieved while still maintaining 85% of specificity (AUC = 0.934). Decreased FA is a common finding in different neuropathies [18, 36–39] and it is considered a non-specific indicator of an overall alteration of fiber coherence which depends on the modification of the direction of water molecules along the axons, possibly related to blood-nerve barrier disruption, impaired axoplasmic flow, intraneural fibrosis, axonal loss. RD is a measure of the diffusion of water molecules perpendicular to the main direction of the axon and is emerging as a biomarker of myelin integrity [16, 17, 40].

Despite the lack of a validated DTI threshold able to differentiate neuropathies with different etiologies, we observed higher RD values of the sciatic nerve in ATTRv-PN compared to diabetic polyneuropathy (1.35 ± 0.15 vs 1.18 ± 0.20) [38], thus confirming the presence of an ultrastructural pattern different from other axonal polyneuropathies. If this finding may reflect a demyelinating component should be disentangled with other approaches but it should be kept in mind that the most common misdiagnosis of ATTRv-PN is chronic inflammatory demyelinating polyradiculoneuropathy (CIDP) [41].

FA and RD significantly correlated with CSA and NSI of the sciatic nerve, with the NIS-LL score and with SNAPs of the sural nerve, thus supporting their possible role as a potential biomarker of ATTRv amyloidosis neuropathy. Notably a significant correlation was also demonstrated with cMAPs and MCV of the fibular nerve and SCV of the sural nerve which are associated with myelin involvement.

The correlation of DTI and neurophysiological data had already been demonstrated in neuropathies of different etiology [18, 36–39, 41–44]. In particular, lower AD values were associated with lower action potential amplitude (axonal polyneuropathy) and higher RD values with slower nerve conduction velocities (demyelinating polyneuropathy). The results of our study suggest a possible wider involvement of nerve structures in ATTRv amyloidosis than in other axonal polyneuropathies but a comprehensive explanation of the link between microstructural nerve alteration and neurophysiological findings is beyond the scope of our study. NSI did not correlate with electrophysiology similarly to T2 relaxometry and proton spin density of the sciatic nerve in a cohort of amyloid light chain (AL) polyneuropathy [35].

Notably, despite inter-scanner variability, lack of standardization of acquisition protocols, and different DTI software for data analysis, the DTI values of the sciatic nerve in our control subjects were comparable with other DTI studies [15, 16, 18]; therefore, the measurement bias may be small enough to not impact the difference between normal vs. pathologic conditions, as demonstrated in previous studies [45].

A theoretical alternative to such a complex methodological approach, based on expensive and not widely available advanced MR imaging techniques, might be represented by high-resolution ultrasound of the sciatic nerve. However, considering the dependency of ultrasound on the examiner’s experience, its limitations in deep localizations such as proximal and mid-thigh, together with the inability to provide functional information beyond the morphological assessment of the size of the nerve and fascicles, this option is likely not very useful and reliable [46].

This study has some limitations. The number of recruited patients is low, especially the number of ATTR-C. Therefore, as in other studies on rare diseases such as ATTRv amyloidosis, investigated with new diagnostic techniques, a power analysis of the minimum sample size has not been applied, theoretically reducing the clinical impact of the study. Nerve conduction studies were conducted in different centers each with their own normative values. A standardized evaluation of autonomic and small fibers function was not performed across the different Centers. The investigation of sciatic nerves was limited to the right thigh, without separate evaluation of tibial and peroneal divisions, although TTR polyneuropathy is a symmetric neuropathy involving both legs. No sural nerve biopsies were available.

Our findings taken together (i.e. enlarged CSA, increased NSI, decreased FA, increased MD and RD) may represent the results of combined pathological processes including: amyloid deposition at proximal sites, increase permeability of blood-nerve barrier secondary to amyloid deposition and damage to vasa nervorum, focal demyelination, axonal loss, alteration of axoplasmic flow, abnormal capillary permeability [47–50].

As a last observation, it is interesting to note that MRN-DTI was able to identify nerve abnormalities 20 years before the presumed age of onset [51, 52] according to the ATTRv amyloidosis Italian registry in 6 of 7 pre-symptomatic carriers [53].

Our preliminary findings confirm the clinical impact of magnetic resonance advanced techniques in the diagnostic workup of ATTRv amyloidosis, however further validation of our results is required in order to establish the role of MRN and DTI in monitoring the disease progression and the response to therapy. As part of an ongoing multicentric Italian study on ATTRv amyloidosis, a longitudinal assessment of the recruited patients with follow-up MRI investigation has been planned.

Considering the limited information about the diagnostic accuracy of DTI in different polyneuropathies, it may be useful as a future perspective, to prospectively evaluate with the same MR imaging approach control groups such as patients affected by inflammatory demyelinating neuropathies (e.g. chronic inflammatory demyelinating polyradiculoneuropathy, CIDP) and axonal neuropathies (e.g. diabetic polyneuropathy).

## Conclusion

**Fig. 6:**
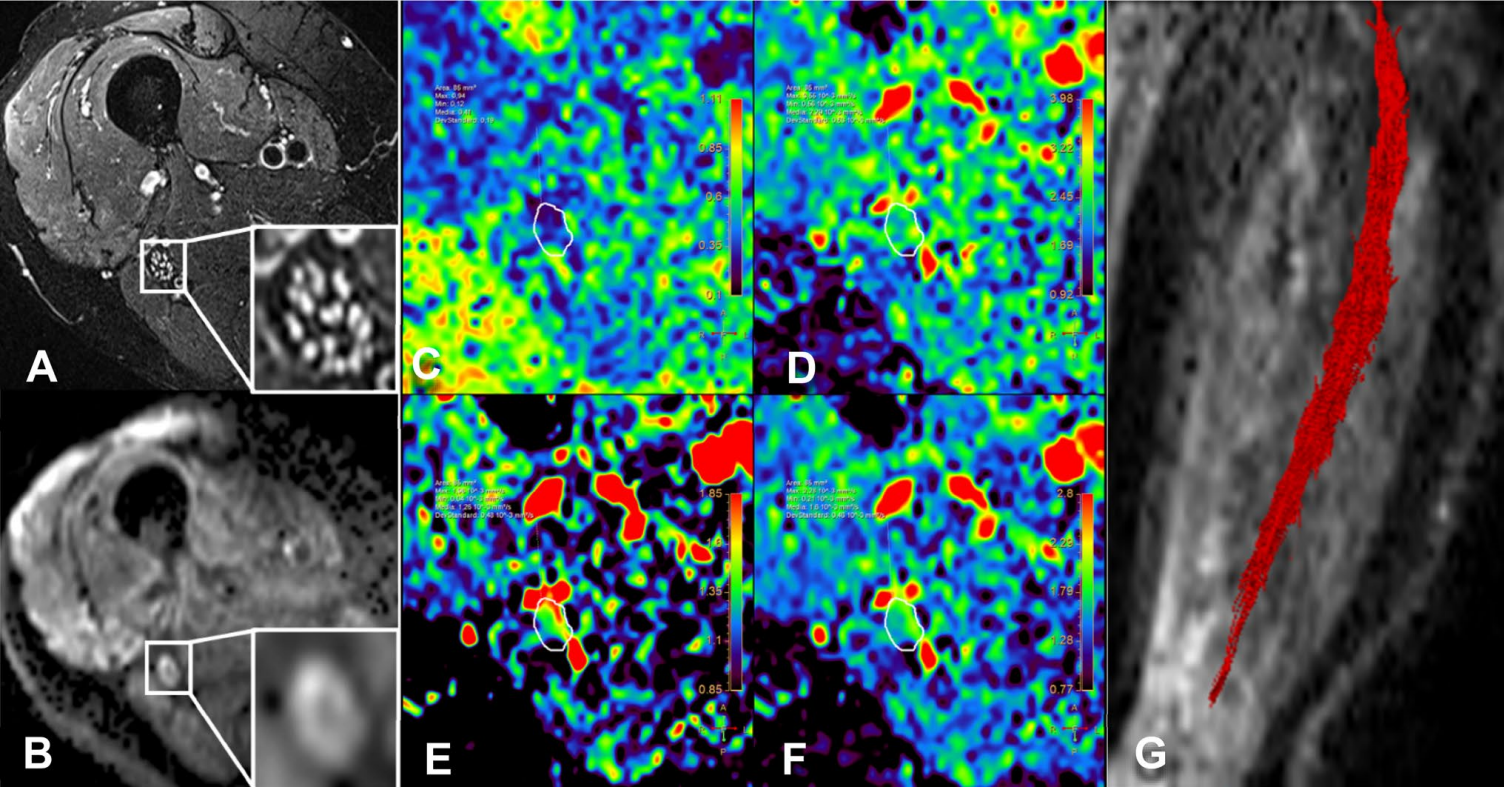
ATTRv-PN. MR Neurography, sciatic nerve at mid-thigh. **B** DWI trace-weighted image (*b* = 700), **C** FA, **D** AD, **E** RD, **F** MD maps, **G** sciatic nerve tractography at thigh. Sciatic nerve CSA 82.7 mm^2^, NSI = 2, FA = 0.41, AD = 2.29 10^–3^ mm^2^/s, RD = 1.26 10^–3^ mm^2^/s, ADC = 1.6 10^–3^ mm^2^/s. Increased signal intensity and size of the sciatic nerve, with significant reduction of FA

**Fig. 7:**
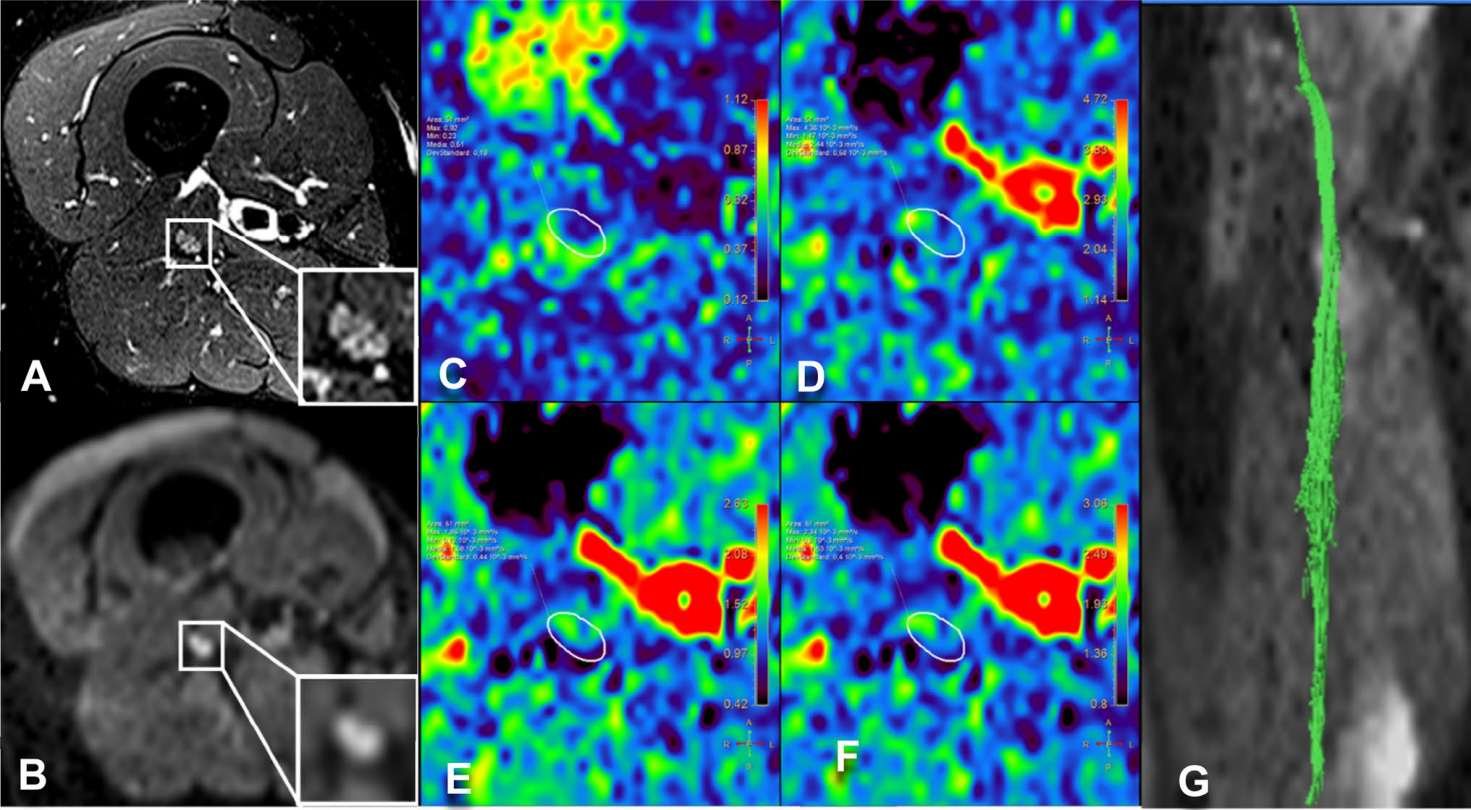
ATTRv-C. MR Neurography, sciatic nerve at mid-thigh. **B** DWI trace-weighted image (*b* = 700), **C** FA, **D** AD, E) RD, **F** MD maps, **G** sciatic nerve tractography at thigh. Sciatic nerve CSA = 42.6 mm^2^, NSI = 1.70, FA = 0.51, AD = 2.44 10^–3^ mm^2^/s, RD = 1.08 10^–3^ mm^2^/s, ADC = 1.53 10^–3^ mm^2^/s. Increased size and signal intensity of the fascicles of the sciatic nerve with no significant changes of CSA and FA

**Fig. 8:**
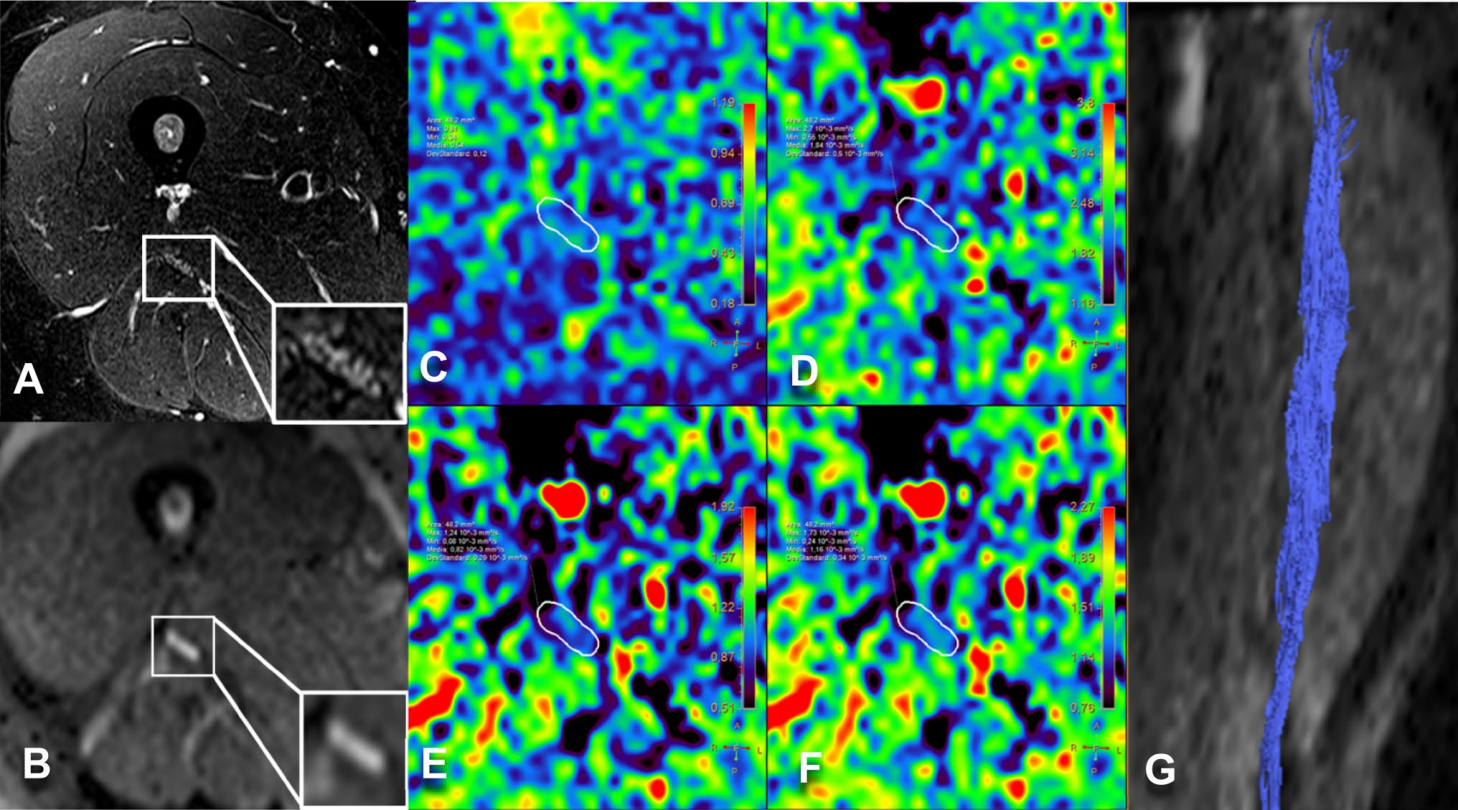
Healthy control. **A** MR Neurography, sciatic nerve at mid-thigh. **B** DWI trace-weighted image (*b* = 700), **C** FA, **D** AD, **E** RD, **F** MD maps, **G** sciatic nerve tractography at thigh. Sciatic nerve CSA 48 mm^2^, NSI = 1.16, FA = 0.54, AD = 1.84 10^–3^ mm^2^/s, RD = 0.82 10^–3^ mm^2^/s, ADC = 1.16 10^–3^ mm^2^/s

A quantitative evaluation of DTI and morphological parameters of the sciatic nerve was able to detect microstructural differences between ATTRv-PN (Fig. 6), ATTRv-C (Fig. 7), and healthy controls (Fig. 8).

The CSA of the sciatic nerve is a general neuroimaging marker of structural abnormalities in neuropathies, unable to differentiate healthy controls from pre-symptomatic ATTRv-C. However, it seems to be useful to differentiate ATTRv-C from ATTRv_PN patients at mid-thigh and distal thigh.

NSI of the sciatic nerve may reflect the presence of impaired axoplasmic flow and perineurial and intraneurial edema and it is unable to differentiate ATTRv-C from ATTRv-PN. Increased NSI instead better differentiates ATTRv-C from healthy controls at proximal and mid-thigh.

Fractional anisotropy (FA) reflects the microstructural integrity of sciatic nerve fascicles and, although not specific for axonal loss, is the most reliable DTI parameter to differentiate pre-symptomatic ATTRv-C from healthy controls at mid-thigh, with the best correlation with NIS-LL and nerve conduction studies.

Radial diffusivity (RD) represents a marker of demyelination and is able to differentiate pre-symptomatic ATTRv-C from healthy controls and ATTRv-PN at proximal and mid-thigh.

The combination of CSA and NSI is able to differentiate ATTRv-C from healthy controls and ATTRV-PN at the distal thigh.

The MRN/DTI measures may represent a sensitive tool to monitor the evolution from the status of pre-symptomatic carrier to patient in ATTRv amyloidosis and also may represent a quantifiable outcome measure in clinical trials.
